# Anatomical Analysis of Thumb Opponency Movement in the Capuchin Monkey (*Sapajus sp*)

**DOI:** 10.1371/journal.pone.0087288

**Published:** 2014-02-03

**Authors:** Roqueline A. G. M. F. Aversi-Ferreira, Rafael Souto Maior, Ashraf Aziz, Janine M. Ziermann, Hisao Nishijo, Carlos Tomaz, Maria Clotilde H. Tavares, Tales Alexandre Aversi-Ferreira

**Affiliations:** 1 Laboratory of Anthropology, Biochemistry, Neuroscience and Primate Behavior, Federal University of Tocantins, Palmas TO, Brazil; 2 Primate Center and Laboratory of Neurosciences and Behavior, Department of Physiological Sciences, Institute of Biology, University of Brasília, Brasilia DF, Brazil; 3 Department of Anatomy, Howard University College of Medicine, Numa Adams Building, Washington DC, United States of America; 4 System Emotional Science, Graduate School of Medicine and Pharmaceutical Sciences, University of Toyama, Toyama, Japan; University of Western Ontario, Canada

## Abstract

Capuchin monkeys present a wide variety of manipulatory skills and make routine use of tools both in captivity and in the wild. Efficient handling of objects in this genus has led several investigators to assume near-human thumb movements despite the lack of anatomical studies. Here we perform an anatomical analysis of muscles and bones in the capuchin hand. Trapezo-metacarpal joint surfaces observed in capuchins indicate that medial rotation of metacarpal I is either absent or very limited. Overall, bone structural arrangement and thumb position relative to the other digits and the hand’s palm suggest that capuchins are unable to perform any kind of thumb opponency, but rather a ‘lateral pinch’ movement. Although the capuchin hand apparatus bears other features necessary for complex tool use, the lack thumb opposition movements suggests that a developed cognitive and motor nervous system may be even more important for high manipulatory skills than traditionally held.

## Introduction

It was traditionally held that no New World primate genera are able to perform precision grips [1], [2]. Nevertheless, more recent investigators have claimed to show evidence of such behaviors in the capuchins [3]–[5]. Functional grips to grab objects using lateral (thenar) sides of the digits have been attributed to a relative capacity to move fingers independently [6]. Anatomical evidence so far does not corroborate this hypothesis [7], [8]. Spinozzi et al. [9] reported that the capuchins grasp food objects in the palm by closing all digits simultaneously, with the thumb flexed parallel with the fingers. They also reported that the capuchins, less frequently, also grab peanuts between a flexed thumb and the palm, or in their thenar and hypothenar eminences. Christel and Fragaszy [4] indicated that although the capuchin monkeys use all digits together for flexion and extension, they were able to perform some form of fine grips.

Capuchins show a variety of grips involving the thumb and the index finger [9], [10]. In such movements, the object is generally grabbed between the lateral (thenar) surfaces of the distal phalanges. Nevertheless, contact between the thumb and the index finger pulps have not been reported in this genus, as is the case for humans and a few other catarrhine genera [2],[11]. This probably stems from the limited ability to rotate the thumb towards other fingers in the capuchins [1]. Napier [2], [12] has described the capuchin thumb as pseudo-opposable mostly due to a lack of a grip using the finger pulps; however, reports of strong lateral (thenar) grips between the thumb and the index finger have led other authors to describe it as ‘lateral opposition’ [4], [6]. Although thumb opposition may not be required for all precision grips strategies, it does grant considerable control while handling fine objects [2]. Any degree of opposition would indicate an important evolutionary convergence with apes and humans, with serious implications on the selective pressures and habits (such as arboreality, terrestrialism), which are thought to have influenced the inception of tool use [13]. Unfortunately, these previous studies, which are based exclusively on behavior, ascribed functional capacities to the capuchin’s hand apparatus in the absence of prior anatomical analyses.

Therefore, we have performed dissections of the capuchin hand, describing the bones, the joints and the muscles involved in hand movements, especially those of the thumb. We have undertaken this morphological investigation to test whether the capuchin hand apparatus is able to support any kind of thumb opposition.

## Materials and Methods

### Ethics Statement

This work was approved by the Institutional Ethical Committee from the Federal University of Goiás (CoEP-UFG 81/2008, authorization from the IBAMA number 15275 for the proceedings detailed here). The details of animal use and welfare were in accordance with the recommendations of the Weatherall report, “The use of non-human primates in research”. In addition to the anatomical analysis of capuchin specimens, we performed simple hand measures of eight human participants in Brazil. These procedures with human subjects were also approved by the Institutional Ethical Committee from the Federal University of Goiás (UFG No 065/2010). All participants signed a letter of informed consent for the procedures.

### Samples

Eight adult cadaveric capuchins (six males and two females) weighing from one to three kilograms were used in this investigation. No animal was killed or euthanized for the purposes of this study: four of them suffered accidental deaths in the woods inside the campus of the Federal University of Goiás (three suffered accidents on the power grid cables, and one was hit by an automobile) and were acquired from the collection of the Anthropology, Biochemistry, Neuroscience and Behavior of Primates Laboratory (LABINECOP) of the Federal University of Tocantins-Palmas-Tocantins (Brazil). The remaining specimens belonged to the Brazilian Institute of Environment and Renewable Natural Resources (IBAMA) archive and were donated to the University of Tocantins for research. All specimens presently belong to the Laboratory of Anthropology, Biochemistry, Neuroscience and Primate Behavior, Federal University of Tocantins, and access to them requires permission from its chairperson.

### Preparation of the Animals for Dissection

All procedures involving the animals were done in accordance with the guidelines of the Brazilian Society of Animal Experimentation (COBEA). After trichotomy with a scalpel blade, the animals were incubated in water at room temperature for 10–12 hours. They received perfusion, by the femoral vein, of 10% of formaldehyde with 5% of glycerin for fixation. The animals were conserved in 10% formaldehyde in covered opaque cubes to avoid the penetration of light and the evaporation of the preservative.

### Dissection and Documentation

Hand dissection was performed on four subjects to expose the muscles and on other four subjects to expose the carpal bones and the trapezium-metacarpal joint. All observations were recorded by digital photography, schematic drawings and annotation. Carpal bones were analyzed based on their longest dimension, irrespective of their size and ratio relative to the carpus. The longest transverse and longitudinal dimensions were taken in straight line and curved configurations. A caliper rule was used directly for straight line measures. In the case of curved surfaces, an inextensible line was used to set the length to be measured with the caliper rule. Hand and digits measurements were performed to calculate shape index (SI), relative digit length index (DI) and the thumb index (TI) using the respective formula below:
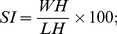


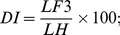


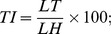
where WH is the width of the hand, LH is the length of the hand, LF3 is the length of finger III and LT is the length of thumb. The statistic analysis (Chi-square) on carpal bones and morphometry was performed using the program StatPlus:mac de AnalystSoft Inc./2009.

The nomenclature of the hand muscles whenever possible follows the one that is used in reputable contemporary human anatomy textbooks [14]. However, we also used the common names designated according to the norm found in Terminologia Anatomica (FCAT) [15].

## Results and Discussion

### The Trapezium-metacarpal Joint

**Figure 1:**
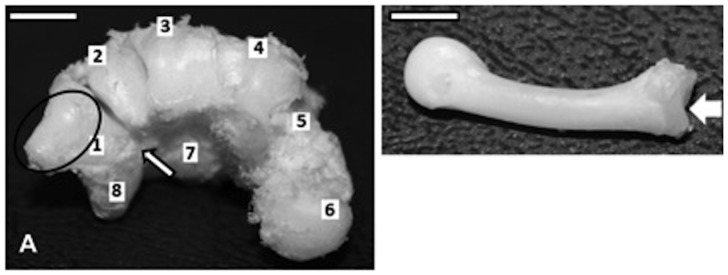
Bones and ligaments in a capuchin left hand. A) surface of carpo-metacarpal joint. The black circle highlights the convex lateral joint surface of trapezium bone with metacarpal; the arrow indicates the central bone; 1) trapezium; 2) trapezoid; 3) capitate; 4) hamate; 5) pyramidal; 6) pisiform; 7) lunate; 8) scaphoid (bar = 4.0 cm). B) Lateral view of metacarpal I. The white arrow indicates the concave surface joint that connects with the trapezium bone (bar = 2.0 cm).

With regards to its articular surface, we found that the capuchin trapezium-metacarpal joint is concave at the base of metacarpal I and convex at the articular face of the trapezium, yielding a 90° angle between them. This arrangement corresponds more closely to a saddle type joint, morphologically (figure 1). Ankel-Simons [16] described it as being closer to a two-axial saddle joint whereas Napier [2] defined it as a hinge type of joint. The depth of the thumb joint surfaces varies among primates, even within genera. A few primates, for instance *Saimiri* (a New World primate), *Tupaia* (a tree shrew) and *Tarsius* (a 'prosimian', or non-anthropoid primate), present a true saddle joint for the thumb whereas marmosets and other prosimians present a shallower joint. In general, however, this joint is less shallow in New World primates when compared with the Old World primates, including the great apes and modern humans [16].

Napier [12] grouped manipulative properties as follows: (1) marmosets and tarsiers present non-opposable thumbs; (2) all prosimians and Cebidae present pseudo-opposable thumbs; (3) Old World primates, including the great apes and modern humans, present truly opposable thumbs; and (4) Hylobatidae present a long opposable thumb. It is interesting to note that the capuchins and the marmosets do not show significant morphological differences in their thumb joint [16], yet they are placed in different groups as regards to their manipulative skills.

Besides the joint surface shapes, other factors are associated with the trapezium metacarpal joint mobility, such as the arrangement of ligaments and other soft tissues [17], which in turn have played an important role in manipulatory behaviors [18]. No analysis of soft tissues (including ligaments) was undertaken in the present work; however, we inspected the bones and joints in detail. The joint surface morphology of capuchin *Sapajus* suggests limitations of certain actions. The trapezium-metacarpal joint surfaces observed in the capuchins indicate that there is either no medial rotation of metacarpal I or that it is very limited. The present data are in keeping with Napier’s view [2], [18] which considers this articulation as being of hinge variety, in functional terms; this would not allow true opposition. In fact, the rotation of the metacarpal I over the trapezium is an essential feature for truly opposable thumbs [12]. Rose [19] indicates that there is rotation of metacarpal I at the thumb joint of the capuchin and other New World monkeys. All synovial joints allow two-dimensional movements, such as rotation and gliding. Nevertheless, it has not been established whether metacarpal rotation, in the case of capuchins, bears any effect on thumb opposition. Also, it is important to note the following regarding the method used to measure rotation: if the test is performed using an external force, there may be a rotation due to the elasticity of soft tissues around the joint. That same movement may not be inferred from a superficial analysis of the actions of internal osteological and arthrological structures involved at the joint.

**Figure 2:**
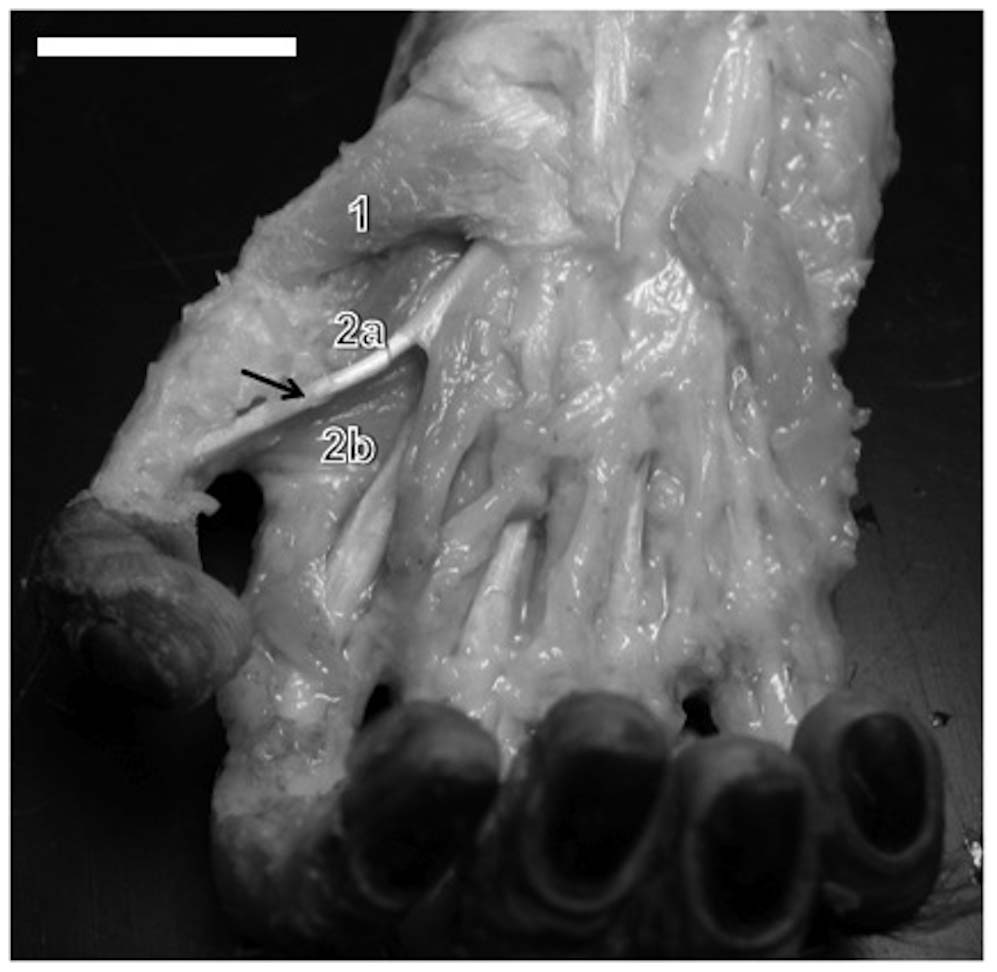
A capuchin right hand. Dissection shows (arrow) the tendon of insertion of digit I long flexor muscle, (1) opponent muscle, and adductor muscle’s (2a) oblique head and (2b) transverse head. (bar = 2.8 cm).

In this sense, it is interesting to notice the opponent muscle of digit I originates from the trapezium bone and the trapezium-metacarpal articulation capsule. It is inserted in the anterior margin of metacarpal I and in the base of metacarpo-phalangeal capsule. It is located underneath the short abductor muscle, with which it shares fibers (figure 2). This muscle exists in most primates, peaking its development in humans according to Swindler and Wood [20]. Napier [18] defined it as rotatory muscle located in a privileged position to act on the trapezium-metacarpal articulation. The different insertion found in capuchin indicates that a more flexing, rather than rotating, action. It will not allow, for instance, the cushion of thumbs distal phalanx to rotate towards the cushion of the other digits. In fact, the flexing action of digit I opponent muscle is more consistent with phylogenetic history since this muscle derives, together with the ‘superficial head’ of the flexor pollicis brevis, from the flexor brevis profundus [21].

### Carpal Bones

**Figure 3:**
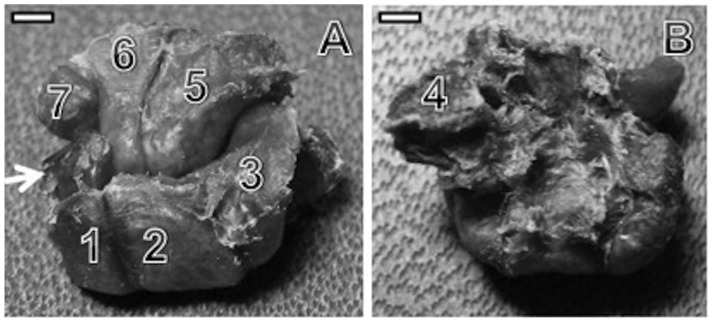
Carpal bones in the right hand of a capuchin monkey. A. Top view of the carpus: (1) scaphoid, (2) lunate, (3) pyramidal, (5) hamate, (6) capitate and (7) trapezoid bones. The arrow indicates the central bone. B. Bottomview of the carpus: (4) pisiform bone. The trapezium bone is not present in this piece (bar = 4 cm).

**Figure 4:**
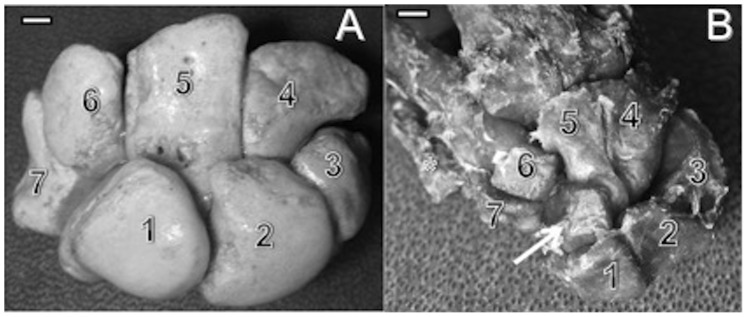
Comparison of human and capuchin carpal bones. A. Carpal bones in the human right hand (top view): (1) scaphoid, (2) lunate, (3) pyramidal, (4) hamate, (5) capitate, (6) trapezoid and (7) trapezium bones (bar = 2 cm). B. Carpal bones in the capuchin right hand (top view). The numbers indicate the corresponding bones of figure 14A. The arrow indicate the central bone and the asterisk * indicantes the metacarpal I (bar = 6 cm). The pisiform bone is not visible in either photographs.

There are nine carpal bones in the capuchin; they are arranged in two rows: four bones at the proximal row and five at the distal row. The same arrangement is found in lemurs and in rhesus monkeys [16]. In Table 1, we compared the measurements between the longest axis of each carpal bone, in any direction, and the longest longitudinal axis of the carpus. The lunate, trapezium, trapezoid and the capitate are proportionally larger in humans; the pisiform and pyramidal bones are proportionally larger in the capuchin, whereas scaphoid and hamate have similar size in both (Chi-square p<0.05). The capuchins show an extra carpal identified as the central bone (“os central”), which is located laterally (thenar aspect) at the proximal portion of the distal row. It is connected distally to the trapezoid, medially to the capitate, proximately to the scaphoid (figures 3 and 4), and palmar-distally to the trapezium. Its longest axis is approximately the same length as the trapezium and the trapezoid. This bone is commonly found in all primates, except that, in apes it usually fuses with the scaphoid prior to birth (e.g., humans and gorillas) or soon thereafter (e.g., chimpanzees) [2].

**Table 1:** Ratio (plus standard deviation) between carpal bones and carpus in capuchin and human (^†^ significant difference; Chi-square, p<0.05).

Bones	Capuchin (longestlongitudinal axisaverage = 1,2 cm)	Humans (longestlongitudinal axisaverage = 3,2 cm)
Scaphoid	0,76(±0,04)	0,79(±0,08)
Lunate^†^	0,52(±0,10)	0,60(±0,07)
Pisiform^†^	0,62(±0,10)	0,45(±0,05)
Pyramidal^†^	0,63(±0,01)	0,57(±0,02)
Trapezium^†^	0,48(±0,06)	0,61(±0,04)
Trapezoid^†^	0,43(±0,02)	0,48(±0,06)
Capitate	0,61(±0,13)	0,66(±0,05)
Hamate^†^	0,71(±0,28)	0,65(±0,07)
Central	0,37(±0,08)	–

The position and characteristics of the capuchin central bone are in accordance with the observations of Schwartz and Yamada [22] for New World monkeys: namely, it is laterally expanded and medially truncated, covering part of the capitate. It is also covered dorsally by the scaphoid, and its distal portion is partially covered by the trapezoid (figure 4).

**Figure 5:**
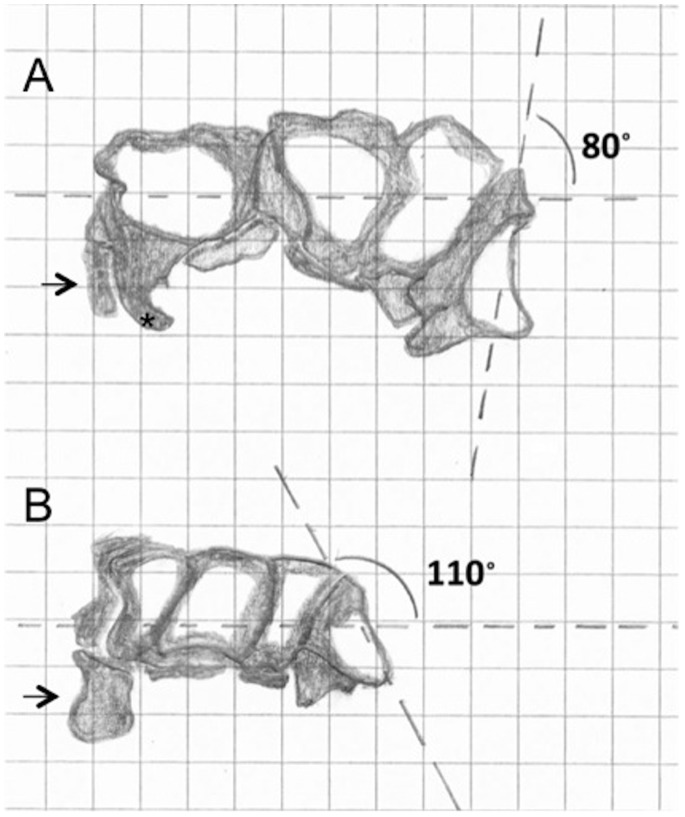
Schematic representation of axis relations in capuchin and humans. Note that the trapezium in humans is located lower relative to the horizontal axis in humans (A) than in capuchin (B). The angle between the carpal plane and the longest trapezium axis is approximately 80° in humans (Napier, 1955) and 110° in capuchins. * indicates the hamate’s hamulus and the arrow indicates the pisiform bone, which is proportionally larger in capuchin.

In fact, the compared relative measures of the capuchin and humans denote differences in the dimensions of the carpals. The absence of the central bone possibly allowed for a few changes in the human trapezium, which is larger and positioned anteriorly. These adaptations also include an 80° angle between the articular face and the plane of the palm (figure 5). Together they provide stronger support and greater mobility of digits I and II [2]. Such mobility is especially important in true thumb opposition. Since metacarpal I is anteriorly located relative to the remaining metacarpals, the opponens and flexor muscles of the thumb adduct and flex it so that the distal phalanx of digit I touches the distal phalanges of the remaining digits [18]. Indeed, the human trapezium is larger and has more shared surface with scaphoid and with metacarpal I relative to all other primates [23]. This aspect is discussed further in this study (table 1).

In the capuchin, the angle between the longest axis and the joint surface of the trapezium and the palmar plane is approximately 110° (figure 5). According to this arrangement, the thumb is nearly in the same axis as the remaining metacarpals, so the opponens and flexor muscles pull it toward the lateral (thenar) side of digit II metacarpal and phalanges. This generates the thumb’s adduction and flexing movements as observed in behavioral studies [4]. Although this movement has been labeled as pseudo-opposition in the capuchins before [6], it does not in fact meet the requirements of the concept of opposition or pseudo-opposition put forward by Napier [2], [12], [18]. Christel and Fragaszy [4] employed the expression ‘lateralized opposition’ for this same movement and adduced studies that supported the existence of some form of “precision” grip in capuchin monkeys [3], [5].

Therefore, it is clear that there is a debate in the literature regarding the name given to thumb movements associated with tool use in the capuchins. Most investigators agree that the capuchin does employ all its digits for gripping movements [4], [9]. This consensus was reached by behavioral researchers *before* detailed anatomical studies had been conducted, i.e. no attempt was made to observe the morphological underpinning of the so-called precise manipulatory behavior in the capuchin [7], [8]. In view of these findings, Napier’s proposal [12] for grouping primates according to thumb mobility would require that the capuchin be given a special, separate grouping (as in the case of Hylobatidae). The pseudo-opposable group as pointed out by Ankel-Simons [16] is very heterogeneous regarding the morphology of the trapezium-metacarpal joint, including, for example, the prosimian *Tarsius* which presents mobility in the metacarpophalangeal joint but not in the carpo-metacarpal joint. It is clear that further studies are necessary to correctly determine the morphological basis and variety of thumb movements in New World primates. Nonetheless, as explained above, the present findings do not warrant the use of opposition or even pseudo-opposition to characterize the capuchin thumb movements during object manipulation.

There is a special problem relating to the nomenclatural designation and the meaning of terms such as lateral opposition or pseudo-opposition as used by anatomists. Terms such as ‘lateral grip’, ‘lateral pinch’ or even ‘lateral crimper’ [14] have been proposed yet not used to ascribe the capuchin thumb movements. Our findings indicate that the capuchin thumb actions may indeed be most accurately described as lateral pinch ‘movement’. Spinozzi et al. [9] indicate that the capuchin performs movements involving the thumb and the lateral (thenar) surface of the distal phalanx of digit II in such a way that the object grabbed is touched by the medial margin of the thumb and lateral side of digit II. Christel and Fragaszy [4] indicate that the capuchin precision grips involve the thumb’s nail or adjacent area, and lateral side of digit II. Both accounts, however, match the lateral pinch movement described by Standring [14] and Napier [2], [12], [18], [24] for human hand movements, but not for the capuchin.

In summary, our description and analysis of the carpal bones, particularly the trapezium, and the lack of medial rotation of the metacarpal I indicate that capuchins are not able to perform true thumb opposition (or pseudo-opposition) as put forward by Napier [2], [12]. Instead, their thumbs exhibit a grip movement more accurately described as lateral pinch, as opposed to ‘lateral opposition’. Data relating the size of hand osteology and the position of the thumb also corroborate this claim.

To compare the relative size of the carpus between capuchin and humans, we measured the longest transversal and longitudinal axes, the lateral curvature of the carpus (with and without the pisiform bone), and the longest axis between the bottom of the carpus and the distal end of the trapezium bone for both species. From these, the following relative measures were derived: (1) longitudinal axis/transversal axis, (2) longest curved axis of the carpus/transversal axis of the carpus (including pisiform bone), (3) longest curved axis of the carpus/transversal straight axis of the carpus (excluding pisiform bone), and (4) longest axis from carpus to trapezium bone/longitudinal axis of the carpus (table 2).

**Table 2:** Ratio (plus standard deviation) between some axes in the capuchin and human wrists (^†^ significant difference; Chi-square, p<0.05).

Ratio	Capuchin	Humans
1^†^	0.72(±0,13)	0.62(±0,04)
2^†^	1.84(±0,04)	1.54(±0,04)
3	1.66(±0,182)	1.76(±0,12)
4^†^	1.0(±0,001)	0.67(±0,08)

Ratios: 1. longitudinal axis/transversal axis; 2. longest curved axis of the carpus/transversal axis of the carpus (including pisiform bone); 3. longest curved axis of the carpus/transversal straight axis of the carpus (excluding pisiform bone); 4. longest axis from carpus to trapezium bone/longitudinal axis of the carpus.

The measures with and without pisiform bone were included due to its increased size capuchin relative to apes [20]. It has for instance its smallest dimensions in humans and among the highest in capuchin (table 1). This augmented size in capuchins is accounted for the greater number of origins of intrinsic hand muscles and insertions from forearm muscles.

The measures performed here indicate that the capuchin hand, relative to the human hand, is longer (ratio 1, table 2), the carpus is narrower and also longer (ratio 3, table 2) if the pisiform bone is not included. These findings are in accordance to the shape index (SI) and the relative digit length (DL) calculated here for capuchin: 26.98 (±1.25) and 60.06 (±1.80) respectively. Napier [2] found SI = 45.1 (±1.20) and DL = 49 (±1.0) for humans. Napier [2] also calculated the DL for other primates: terrestrial specialists (baboons, in this case) presented DL = 43 (±1.0), an arboreal specialist showed DL = 55 (±3.0) and prosimians DL = 59.5 (±4.5). Combined with these findings, the results shown in table 2 suggest that capuchins are closest to prosimians and arboreal specialists regarding digital length similarity.

The human thumb is comparatively longer but the remaining digits are shorter and less curved; also the palm of the hand and distal phalanges are wider [17]. The thumb shows complete opposition and its longer length, relative to digit II, allows full finger cushion contact between the thumb and the remaining digits [2]. These features may be considered the more derived characteristics within primates leading to thumb opponency.

Another important finding is that the emergence of metacarpal I is more proximal and anterior in the human hand than in capuchin (ratio 4, table 2), due to differences in the trapezium bone and central bone’s amalgamation in the carpus. Accordingly, the thumb index (TI) calculated here yielded 55.34 (±4.20) for the capuchin specimens and 62.8 (±4.0) for humans, which indicates a larger human thumb as compared to capuchin.

The human hand, therefore presents a short and wide carpus, which is indicative of more derived hand, whereas the high DI found in capuchins indicates less derived characteristics, including the presence of the long fingers [2]. Longer fingers hinder oppositional movement, preventing finger cushion contact between them.

### The Disposition of the Capuchin Thumb

**Figure 6:**
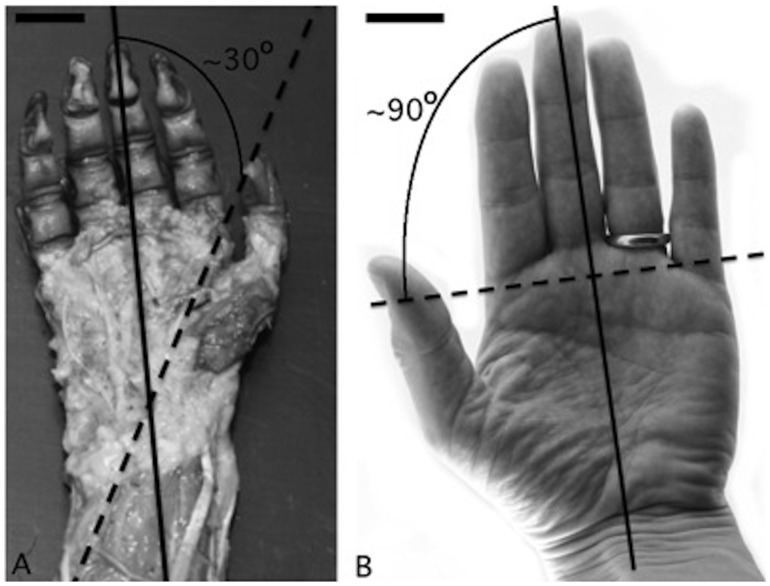
Nail-pulp and medial axes’ angle comparison. (A) Photograph of a capuchin’s right hand, dissected (bar = 0.5 cm) and (B) human left hand (bar = 1.2 cm). Note that the angle formed by the crossing of the axes is of approximately 30° in capuchins and approximately 90° in humans.

Thumb disposition relative to the other digits and the palm of the hand was compared between human and capuchin hand. There is a clear visual difference between humans and capuchins regarding the position of the thumb relative to the other digits of the hand. To specify this arrangement, the relative angle between the medial axis of the hand and the ‘nail-pulp’ axis of the thumb’s distal phalanx were measured (figure 6). For this measurement, the hands were photographed in a semi-flexed position and the orientation of nail-pulp axis was determined as a line with a 90° angle relative to the distal phalange’s longitudinal axis.

The nail-pulp axis was projected over the palm to measure the angle relative to the medial axis. In humans, the crossing of these axes takes place near the base of the proximal phalanx of digit III, i.e. in the distal portion of metacarpal III. In capuchins, on the other hand, this crossing is found lower, i.e. in the proximal portion of the hand where the intrinsic hand muscles originate (figure 6). The angle created by the nail-pulp axis and the medial axis indicates the relative direction where the pulps of the opposing digits face, i.e. it is a measure of the thumb’s medial rotation. In this sense, capuchins present an angle of approximately 30° whereas the angle in the human hand is approximately 90° (figure 6). These data further indicate that thumb rotation, in which the pulp of the thumb faces the medial axis in a right angle, is a feature found in humans but not capuchins. In fact, the flexor muscles in the human thumb bring the distal phalanx pulp closer to hand median axis, facing the lateral side of the hand. In the capuchins, on the other hand, the same movement brings the thumb towards the lateral (thenar) side of the hand; however, in this position the thumb pulp does not face the lateral aspect of the hand.

Another important difference between the movements of the capuchin and human thumb is with reference to its position relative to the plane of the palm. In humans, the articular face of the trapezium is more anteriorly rather than laterally positioned relative to the palm. The opposite is observed in the capuchins: the trapezium is more laterally positioned in its articular face (figure 5). The arrangement in humans allows the flexor muscles to pull the longitudinal axis of the thumb across the median axis of the hand, i.e. the thumb moves anteriorly (palmar aspect) and medially over the palm. In capuchins, the lateral (thenar) position of the trapezium relative to the hand hinders this movement due to the bones and the other tissues in the thenar face of the hand.

## Conclusions

Taken together, our results indicate that the capuchin hand does not have the morphological characteristics that allow true thumb opposition to occur. The opposition movement of the thumb has been long associated with tool use and high cognitive skills in Old World primates. This association is supported by observations of complex use of tools in the great apes [2], [12], [17], [25], [26]. In line with this reasoning, the capuchins were presumed to have some capacity for genuine precise manipulation after several reports of tool use in the wild and in captivity [27]–[30]. These assertions were also based on detailed analysis of manual behavior performed by capuchins in captivity [4], [9]. Nonetheless, this assumption was accepted despite a lack of prior careful anatomical analysis of the forelimb, especially its hand, in capuchins.

The present work does not, however, discuss the merits of such findings regarding the manual behavior observed in capuchins; rather, it is concerned with the specific issue of opposition movements, which have been reported in capuchins in the wild or in captivity. The results shown here do not support the claim that capuchins engage in manipulatory behavior based on genuine thumb opposition. The capuchin manipulates objects using lateral pinch type of opposition. So far such grips were associated with thumb adduction, and possibly for that reason, the capuchin thumb movement was referred as lateral opposition or pseudo-opposition. Indeed, the lateral pinch movement employed by the capuchin to solve manual and cognitively demanding tasks [9] is occasionally observed in humans during manual activities that require high degree of precision, such as writing with a pen or a pencil. Despite the morphological features necessary for high manipulatory skills present in the capuchin hand apparatus, the lack of thumb opponency in capuchins may require varied cognitive strategies [9], [10] and well-developed motor system [31] during complex tool use.
